# Optimizing Computed Tomography for Detection of Pulmonary Thromboembolism in Patients With Fontan Circulation

**DOI:** 10.7759/cureus.8326

**Published:** 2020-05-28

**Authors:** Sohab Radwan, Gauravpal S Gill, Amre Ghazzal, Christopher Barnett

**Affiliations:** 1 Internal Medicine, MedStar Washington Hospital Center, Washington, DC, USA; 2 Cardiology, MedStar Washington Hospital Center, Washington, DC, USA

**Keywords:** fontan, pulmonary embolism, computed tomography

## Abstract

Congenital heart disease (CHD) patients who have undergone the Fontan procedure or one of its variants usually have altered vascular anatomy. Consequently, this poses a challenge when diagnosing pulmonary thromboembolism (PTE) with computed tomography (CT). Detailed review of the type of surgery performed and the person’s individual anatomy beforehand can help in choosing the appropriate diagnostic CT modality and technique. It would also help reduce false-positive and false-negative test results that would otherwise result in unnecessary anticoagulation, as well as avoid needless radiation exposure and additional cost, respectively.

## Introduction

The Fontan procedure, with its many variations, aims to shunt systemic venous blood directly into the pulmonary circulation and therefore bypassing the right ventricle. It is mainly indicated for congenital heart disease (CHD) patients with a single ventricle physiology such as hypoplastic left heart syndrome and tricuspid or pulmonary atresia, with an intact intra-ventricular septum [1]. Due to this altered vascular anatomy, pulmonary thromboembolism (PTE) is a known complication in patients with a single ventricle physiology with Fontan circulation, and has been reported to occur in 1%-33% of this population [2]. This is believed to be multifactorial in etiology, including endothelial damage, sluggish blood flow, as well as the type of Fontan procedure done and conduit used all appear to play a role in pathophysiology [3,4]. As a result of this altered vascular anatomy, diagnosis of PTE, specifically with monophasic computed tomography (CT), may be challenging. Both false-positive and false-negative test results have been reported. False-positive results may lead to inappropriate anticoagulation, and false-negative results may lead to unnecessary radiation exposure and cost. We hereby present a case of a patient with Fontan circulation and demonstrate the technical difficulties associated with diagnosing PTE in this population.

## Case presentation

**Figure 1:**
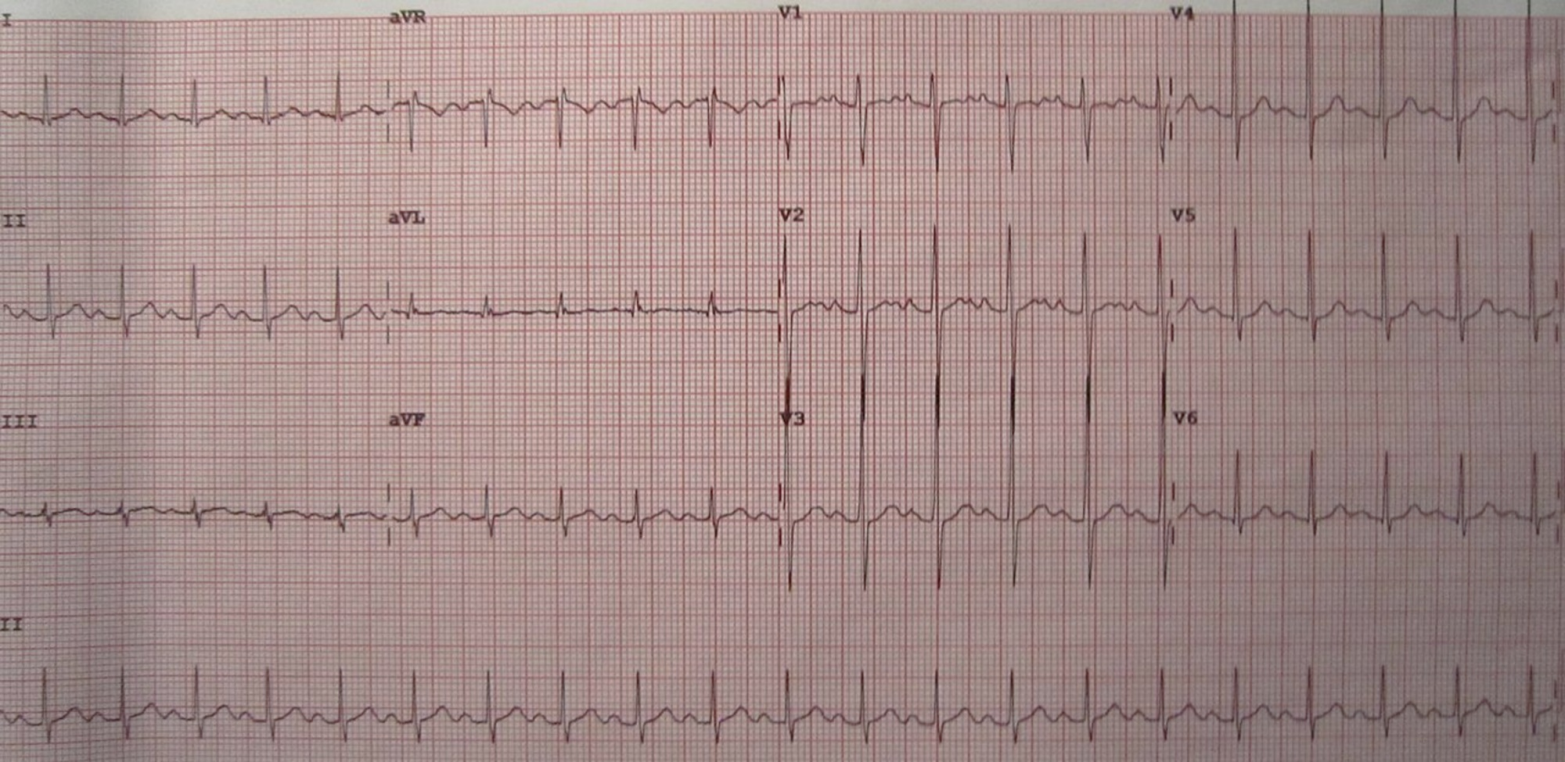
Electrocardiogram demonstrating sinus tachycardia.

**Figure 2:**
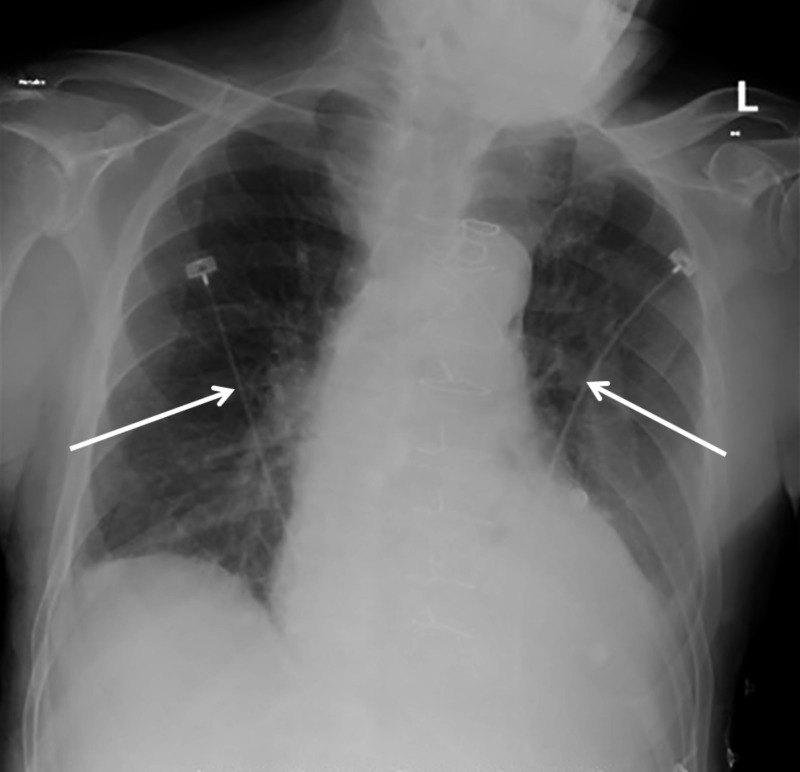
Chest radiography demonstrating pulmonary venous congestion.

A 26-year-old male with a past medical history of dextro-transposition of the great vessels, pulmonary atresia and hypoplastic left heart syndrome, who underwent modified Fontan procedure (total cavopulmonary connection; superior vena cava (SVC) is connected to the right pulmonary artery (PA) and the inferior vena cava (IVC) to the left PA), presented with left-sided pleuritic chest pain and dyspnea on exertion of one-week duration. His vascular anatomy was not known at the time of presentation. Vital signs included a blood pressure of 117/58 mmHg, a heart rate of 112 beats per minute and a respiratory rate of 26 breaths per minute with an oxygen saturation of 84% on two liters of oxygen via nasal cannula. An electrocardiogram (Figure 1) was only significant for sinus tachycardia, and a chest X-ray (Figure 2) revealed pulmonary venous congestion.

 

**Figure 3:**
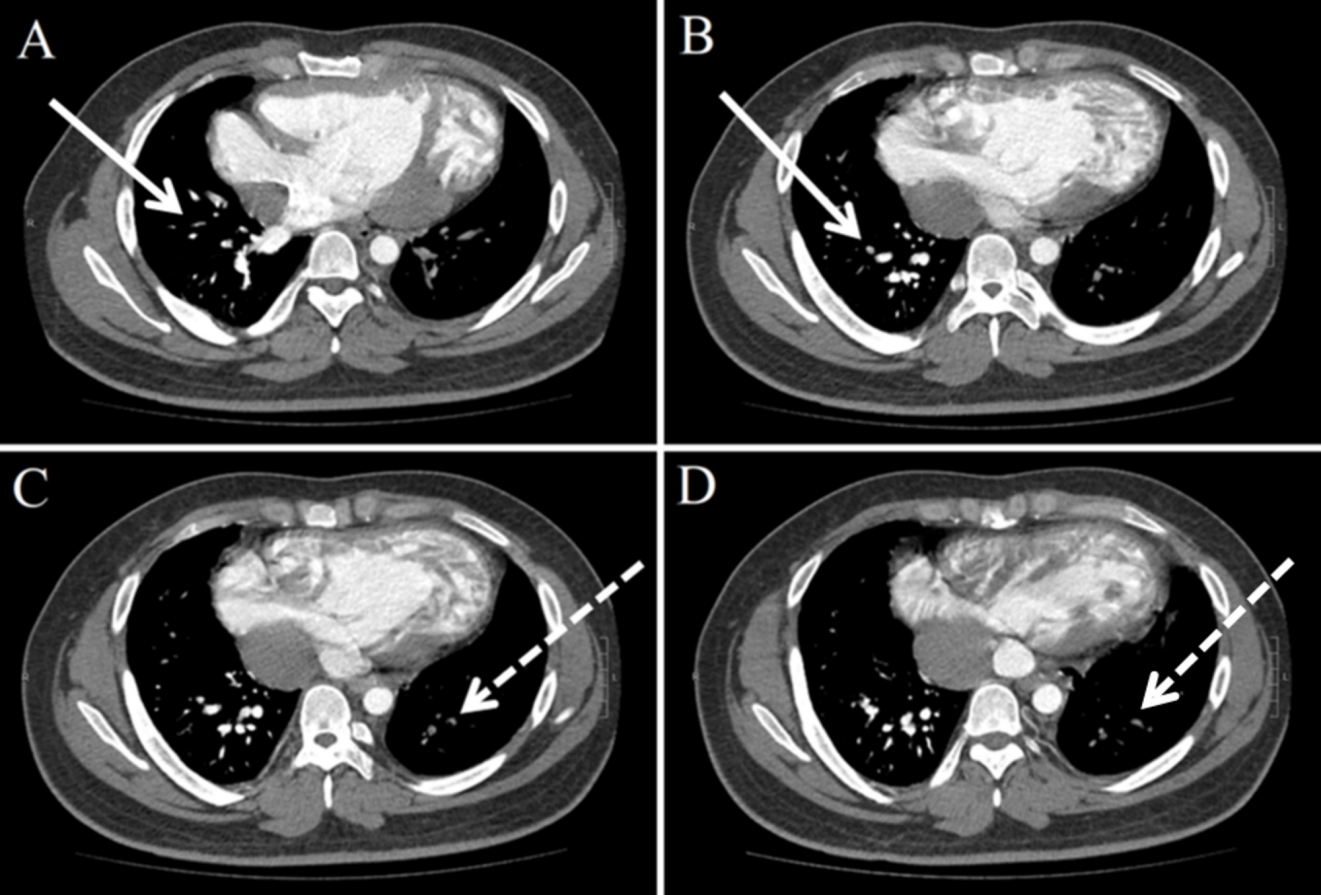
Axial CT pulmonary angiogram demonstrating SVC to be contiguous with the right PA with clear opacification (solid arrow line in panes A and B) and also shows IVC to be contiguous with the left PA, but without any opacification (dotted arrow line in panes C and D). SVC: superior vena cava; PA: pulmonary artery; IVC: inferior vena cava

Due to concern for PTE in the setting of the Fontan procedure, chest CT angiography (iodinated contrast administered via the right upper extremity) was obtained, which demonstrated the SVC to be contiguous with the right PA with a small amount of contrast in vessel, and also showed the IVC to be contiguous with the left PA, however, without any contrast inside (Figure 3).

As a result, evaluation of the PAs for PTE was non-diagnostic. Due to a high clinical suspicion for PTE, anticoagulation with enoxaparin was initiated. The patient also received intravenous diuretics for volume overload with subjective improvement in symptoms within 24 hours of presentation. A ventilation/perfusion lung scan was performed approximately 48 hours after presentation, with radioactive tracer injection in both the upper and lower extremities, and demonstrated a low probability diagnosis of PTE. Enoxaparin was therefore discontinued.

## Discussion

This case illustrates the technical difficulties associated with diagnosing PTE by monophasic CT in patients with Fontan circulation or one of its variants. The first pitfall was not being able to visualize the left PA due to contrast agent administration only via the upper extremity. Given the altered vascular anatomy, contrast agent should also be administered via the lower extremity to reach the left PA in patients with similar Fontan anatomy.

Second, injection of contrast agent via both the upper and lower extremities may be non-diagnostic. Due to absence of a pre-pulmonary pump, Fontan blood flow is slow which may cause a mixture of opacified and unopacified blood to resemble a thrombus, especially if contrast agent administration timing between the upper and lower extremities is not coordinated [5]. This can therefore be challenging to medical providers and radiologists. To allow homogeneous enhancement of the right and left PA and avoid detecting an apparent filling defect due to unopacified blood rather than a true thrombus, simultaneous injection of contrast agent via the upper and lower extremities, in addition to acquiring CT imaging in early and delayed phases, has been suggested [6]. This technique is called the dual injection dual phase technique [6]. An alternative to CT is magnetic resonance angiography, which is also effective for evaluating the Fontan circulation for PTE, but may pose a challenge to medical providers if patients are clinically unstable as it is time inefficient [5].

## Conclusions

Prior to evaluating for PTE in patients with Fontan circulation, acquiring a detailed review of the type of surgery performed is vital. This is especially important to evaluate for any potential vascular anatomic variations that may affect the choice of which extremity to use for contrast agent administration. This would also reduce unnecessary radiation exposure as well as cost.
